# Impact of the Paper by Allen and Humphreys (1979) on Anti-Tick Vaccine Research

**DOI:** 10.3390/pathogens11111253

**Published:** 2022-10-28

**Authors:** Consuelo Almazán

**Affiliations:** Immunology and Vaccines Laboratory, Facultad de Ciencias Naturales, Universidad Autónoma de Querétaro, Queretaro 76140, Mexico; c_almazan_g@hotmail.com

**Keywords:** host resistance, anti-tick vaccines

## Abstract

The classic paper by Allen and Humphreys “Immunisation of guinea pigs and cattle against ticks” Nature, 1979, 280: 491–493 led to a surge in the development of tick vaccines as a nonchemical method for prevention of tick infestations in susceptible hosts living in tick-endemic regions. Although observations of host resistance to ticks had been documented since the beginning of the last century, it was not until publication of this paper that the proof of concept of anti-tick vaccines was developed. The described experimental methods directly impacted further investigations on the discovery and evaluation of new anti-tick vaccines.

## 1. Introduction

Tick immunity or host resistance to ticks was known prior to the classic paper by Allen and Humphreys [1]. In 1918, Johnston and Bancroft documented that cattle became resistant to subsequent infestations by the Australian cattle tick *Rhipicephalus (Boophilus) australis* Fuller, 1899 (Acari, Ixodidae), suggesting an immune response to the substances inoculated during tick feeding [2]. In 1936, Ross observed that dogs living in endemic regions of *Ixodes holocyclus* Neumann, 1899 (Ixodida, Ixodidae) rarely suffered from tick paralysis and hypothesized that this may be due to immunity acquired after several tick bites [3]. Then, in 1939, Trager reported that *Dermacentor andersoni* Stiles, 1908 (Ixodida, Ixodidae) larvae fed on naive guinea pigs achieved an engorgement of 80%, but subsequent infestations only achieved 10% of engorgement [4]. The same year, this author published the results obtained from a series of experiments performed on *D. variabilis* Say, 1821 (Ixodida, Ixodidae) fed on laboratory animals [5]. Based upon these results, it was concluded that (a) one infestation of *D. variabilis* in guinea pigs and rabbits induced immunity preventing larval engorgement; (b) two or three infestations were needed for mice to become resistant to tick larvae; (c) repeated infestations of guinea pigs reduced the consumption of blood by ticks; and (d) immunity to ticks could be induced by subcutaneous inoculation of larval extracts or transferred by intraperitoneal inoculation of the hyperimmune serum obtained from guinea pigs after several tick infestations. This was the first time that immunization against tick infestation was demonstrated. In 1979, the publication by Allen and Humphreys [1] confirmed these results in laboratory animals, extended the concept for use in cattle, and demonstrated that immunological protection against ticks was feasible in production animals. This review aims to emphasize the contributions and the impact of this paper on anti-tick vaccine research more than four decades after its publication.

## 2. Discovery

Before the classic paper by Allen and Humphreys (1979) [1], two papers on the observations and findings in *D. andersoni* fed on laboratory animals were published [6,7]. In the first publication [6], Allen showed that resistance to *D. andersoni* larval infestations was acquired, as demonstrated by the obtention of fewer engorged larvae after feeding on guinea pigs infested for a second time. The role of the immune system was supported by further experiments, which showed that this resistance was prevented by using methotrexate, an immunosuppressant. In a second publication [7], Wikel and Allen documented the passive transfer of tick resistance by showing that the weight of *D. andersoni* larvae engorged on hosts inoculated with immune serum was slightly lower in the controls, suggesting that humoral factors contributed to host resistance.

**Figure 1:**
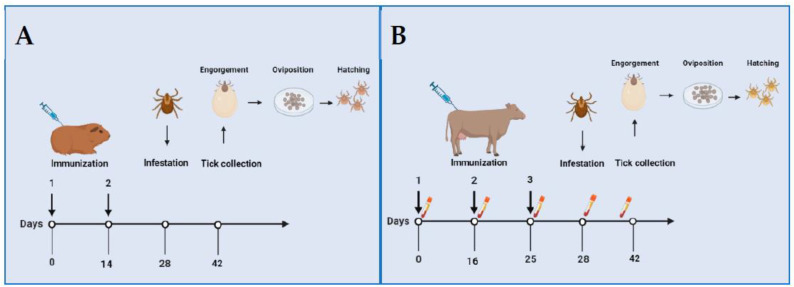
Schematical representation of experiments of immunization against *Dermacentor andersoni* performed by Allen and Humphreys (1979). (**A**) Three groups of guinea pigs were immunized at days 0 and 14 with *D. andersoni* crude extracts from gut and reproductive organs and all internal organs. (**B**) Two groups of 4 Hereford-cross calves were immunized at days 0, 16, and 25 with crude extracts from gut and reproductive organs. Serum samples were obtained at days 0, 16, 25, 28, and 38 for immunodiffusion. Guinea pigs were infested with 4 couples and cattle with 30 males and 100 females of *D. andersoni* adult ticks at day 28. Engorged ticks were collected after two weeks of feeding. Efficacy of immunization was evaluated based on weight of engorged ticks, oviposition, and hatching. Created with Biorender.com.

The paper by Allen and Humphreys [1] described, for the first time, experimental trials of immunization and tick challenge in two separate experiments using laboratory and large animals. The first experiment is represented in Figure 1A. Three groups of guinea pigs were intradermally immunized on days 0 and 14 with 1.2 mg/kg of *D. andersoni* crude extracts emulsified in Freund’s Complete Adjuvant (FCA) or FCA alone. The first group received an extract of gut and reproductive organs (antigen I), the second received an extract of all internal organs (antigen II), and the control group (C) received FCA alone. Each animal was infested with four couples of *D. andersoni* ticks on day 28, and the ticks were allowed to feed for two weeks, when engorgement was evaluated by the weight of each tick. Females were incubated in humidity chambers; and oviposition was evaluated by the weight of egg masses and by counting the number of larvae hatched at 35 days of incubation. These results indicated that antigen I significantly reduced mean egg weight, antigen II blocked oviposition, and both antigens blocked larval hatching. The second experiment is represented in Figure 1B. Two groups of four Hereford-cross calves were used. Calves from group 1 were intramuscularly immunized with 67 mg of antigen I emulsified in Freund’s Incomplete Adjuvant (FIA) on days 0 and 16, followed by a similar dose of antigen without adjuvant on day 25. Calves in group 2 (control) received FIA alone. Each calf was infested with 30 males and 100 females of *D. andersoni* on day 28. The ticks fed on calves from group 1 had a significant reduction in weight, oviposition, and hatching. Immunodiffusion studies on the sera obtained from immunized calves at 0, 16, 25, 28, and 38 days revealed single precipitating bands with the antigen and strong multiple bands on day 38, supporting the hypothesis of immune system activation.

## 3. Impact on Anti-Tick Vaccine Research

Allen and Humphreys [1] hypothesized that immunization against the one-host tick *Rhipicephalus (Boophilus) microplus* would be more effective than in three-host ticks because engorgement of the three developmental stages occurs on the same immunized animals, thus increasing the chance of antibody uptake with blood meals resulting in host protection. This, together with previous observations by Johnston and Bancroft [2], encouraged an investigation by Australian researchers working at the Commonwealth Scientific and Industrial Research Organization (CSIRO). These studies led to the discovery of the first antigen to protect cattle against tick infestations, of which the experimental details were documented in a series of scientific papers published in 1986 [8,9,10]. Briefly, immunization of cattle with purified crude extracts from *R. microplus* females induced protection against infestation with *R. microplus* larvae, resulting in 70% and 90% reductions in the adult tick population in two experimental trials [8]. Histological studies of ticks collected from vaccinated cattle showed a gut distention in ticks with blood leakage into hemolymph. Since the tick gut cells were ruptured, it was suggested that the protective antigen was located on the plasma membranes of these cells [9]. Affected ticks showed red coloration, up to 60% of females and males presented with damaged guts, and affected females failed to engorge or lay eggs [9]. The damage to affected ticks was hypothesized to be due to an antibody response directed to a “concealed tick antigen”, so called because its location was out of reach of the host immune response [10]. This concealed antigen was named Bm86, because of the tick species and the year it was identified; is an 89 kDa membrane-bound extracellular glycoprotein, located on the surface of the *R. microplus* tick’s gut cells [10,11]. The recombinant protein rBm86, expressed in *Escherichia coli*, was used in the formulation of the first vaccine produced against ticks [12]. The production and commercialization of Bm86 under the name TickGARD started in 1994, in Australia [13]. The same year, the Bm86 antigen was isolated and expressed in the yeast *Pichia pastoris* by researchers at the Center for Biotechnology and Genetic Engineering, in Cuba [14]. This recombinant antigen was commercialized in Latin American countries under the name Gavac [15]. The cost and the need for a vaccine dose every three months made anti-tick vaccines unpopular with Australian farmers. Thus, TickGARD was removed from the market in the late 1990s, reintroduced in 2002, and, finally, removed again a few years later because sales represented only 4% of acaricide sales [16]. Currently, Gavac is the only tick vaccine available. More than 3 million cattle have been vaccinated with Bm86, since its introduction on the market [17].

The publication by Allen and Humphreys [1] has greatly impacted the scientific research on anti-tick vaccines. In the two decades following its publication, most of the scientific articles published on tick vaccines reported on molecular characterization, elucidation of the role of Bm86, and its efficacy on tick control [11,12,13,14]. Investigation into the immune response in laboratory and natural hosts infested with tick species other than *R. microplus* has also expanded, as noted by the increase in scientific publications. A search on PubMed (https://pubmed.ncbi.nlm.nih.gov, accessed on 22 September 2022), using the keywords “ticks” and “anti-tick vaccines” during the period of 1979 to 2022, yielded 235 scientific papers. From these, reviews were eliminated, and only 195 papers were considered. From these selected papers, 33% corresponded to *R. microplus*, followed by *I. scapularis, Haemaphysalis longicornis, Amblyomma americanum*, and *I. ricinus* with 24%, 21%, and 2%, respectively.

The discovery and use of Bm86 as an anti-tick vaccine demonstrated that immunological control of ticks was a cost-effective, environmentally friendly, and harmless strategy in comparison with chemical control [18]. Therefore, efforts from different research groups focused on the identification of new protective antigens for vaccine development against not only *R. microplus* but also other ticks of medical and veterinary importance such as *Ixodes* spp., *H. longicornis*, and *A. americanum*. The most relevant antigens discovered after the publication of the paper by Allen and Humphreys [1] are grouped in Table 1. Variations in the efficacy of Bm86 in vaccinated cattle from different geographical regions have been observed [19,20,21]. The low or null efficacy in vaccination with Bm86 in South America encouraged the isolation and evaluation of Bm86 homologues. Bm95, obtained from an Argentinian *R. microplus* strain, showed protection against South American tick strains, suggesting that a broad-spectrum vaccine could be obtained by the inclusion of antigens from different geographical tick strains [21]. Bm86 orthologues were also investigated, and their efficacy against *R. microplus* and *R. annulatus* was evaluated. Interestingly, more efficacious results were obtained with these orthologues (Ba86 and Bm86) against *R. annulatus* than against *R. microplus* [22]. In another study, the immunization of cattle with Haa86 reduced the infestation of *Hyalomma anatolicum* larvae to 67% [23].

Other identified tick antigens in *R. microplus* include aquaporins and metalloproteases. Aquaporins are channels that regulate transportation across cell membranes. Immunization of cattle with a recombinant aquaporin (RmAQP1) showed 75% and 68% efficacy against *R. microplus* in two pen trials [24]. Metalloproteases are multifunctional proteins involved in blood-meal-related functions. The vaccination of cattle with a recombinant metalloprotease (BrRm-MP4) was tested against *R. microplus*, with an overall protection of 60% [25].

Investigation into other potential protective antigens conducted in *R. appendiculatus* resulted in the identification of 64P, a putative cement protein involved in attachment and feeding [26]. Cross reactivity of 64P with *R. sanguineus*, *I. ricinus*, *A. variegatum*, and *R. microplus* was shown. Evaluation of this protein as a vaccine preparation against *I. ricinus* in rabbits resulted in 18–64% and 5–60% mortality of nymphs and adults, respectively [27]. In addition, 64P was shown to react against exposed antigens and protected mice from transmission of tick-borne encephalitis virus (TBEV) by *I. ricinus*, suggesting the feasibility of using anti-tick vaccines to protect against tick-borne pathogens [28].

**Table 1:** Relevant identified tick vaccine antigens evaluated in immunization and tick challenge trials, from 1979 to date.

Tick Species	Antigen	Efficacy	References
* Dermacentor andersoni *	Internal organ tick extracts	Reduction on tick weight, oviposition, and hatching of *D. andersoni* fed on guinea pigs and cattle	[1]
* Rhipicephalus (Boophilus) microplus *	Bm86	Reduction of 70–90% of *R. microplus* tick population in cattle	[8,9,10,12,14]
	Bm95	Reduction of 58% and 89% of *R. microplus* Argentinian and Camcord strains, respectively	[21]
	Aquaporins	75% and 68% efficacy against *R. microplus*	[24]
	Metalloprotease, rBrRm-MP4	Overall efficacy of 60% against *R. microplus*	[25]
* R. (B.) annulatus *	Ba86 orthologue	Efficacy of 83% and 71.5% against *R. annulatus* and *R. microplus*, respectively	[22]
* Hyalomma anatolicum anatolicum *	Bm86 orthologues	Reduction of 69.7% of *H. anatolicum* larvae	[23]
* R. appendiculatus *	Glutathione S-transferase, GST	Efficacy of 67% against *R. appendiculatus.* No efficacy against *R. sanguineus* Efficacy of 43.69% against *D. marginatus* fed on rabbits	[29,30,31]
	Attachment cement protein, 64P	18–64% mortality of nymphs and 5–60% mortality of adults fed on immunized rabbits Protects mice against TBEV infection	[26,27,28]
* Haemaphysalis longicornis *	P0, ribosomal peptide	Overall efficacy of 90% against *R. sanguineus* fed on rabbits and 96% against *R. microplus* in cattle	[32,33,34]
* Ixodes scapularis *	Subolesin	Efficacy of 71%, 63%, and 58% against *I. scapularis* in mice, rabbits, and sheep, respectively Efficacy of 51% and 60% against *R. microplus* and *R. annulatus*, respectively	[35,36,37]
	Salp15	50% protection of mice from *Borrelia burgdorferi* infection	[38,39]
* I. ricinus *	Ferritin	Efficacy of 64% and 72% against *R. microplus* and *R. annulatus*, respectively	[40,41]
	Microbiome Enterobacteriaceae	Significant mortality of *I. ricinus* nymphs fed on mice	[42,43]

Subolesin is a tick-vaccine candidate identified in a cDNA library from *I. scapularis* embryos that is involved in tick feeding and fertility [35]. Other studies concluded that subolesin is involved in gene expression and several cellular pathways [44,45]. When *I. scapularis* larvae, nymphs, and adults were fed on subolesin-immunized mice, rabbits, and sheep, efficacies of 71%, 63%, and 58%, respectively, were obtained [36]. In subolesin-immunized cattle, efficacies of 51% and 60% against *R. microplus* and *R. annulatus*, respectively, were obtained [37]; and the efficacy of vaccination against *R. microplus* increased up to 67% in cattle immunized with a subolesin-derived peptide [46].

Glutathione S-transferases (GSTs) are enzymes with a function in the detoxification of xenobiotic compounds such as drugs and pesticides, and an increase in the activity of GSTs is associated with resistance to synthetic pyrethroids [29]. GSTs are required for heme processing and detoxification during tick blood meals [29]. When a recombinant GST was evaluated as a vaccine against *R*. *appendiculatus* in rabbits, a protection level of about 65% was obtained, but no protection against *R. sanguineus* was demonstrated [30]. In another experiment, vaccination of rabbits with a GST and challenged with *D. marginatus*, resulted in an overall efficacy of 43.69% [31].

Ferritin (FER2), discovered in *I. ricinus*, is a protein with a role that is essential in iron metabolism and was shown to affect tick feeding [40]. FER2 has been evaluated in vaccination trials against *R. microplus* and *R. annulatus* in cattle, with efficacies of 64% and 72%, respectively [40]. However, when used for vaccination against *I. ricinus* in cattle, significant results were not obtained [41].

Functional studies on *H. longicornis* resulted in the identification of Protein 0 (P0), a ribosomal protein with a principal role of the regulation of transcription, which is required for blood ingestion and tick viability [32]. A 20 aa peptide from the P0 protein was evaluated as an anti-tick vaccine against *R. sanguineus* in rabbits, with an efficacy of 90% [33]. Subsequent studies showed 85% efficacy against *R. sanguineus* and *R. microplus* in dogs and cattle, respectively [34].

Salp15 is a 15 kDa protein from tick saliva that was identified in *I. scapularis*, which was associated with immunosuppression by inhibiting cellular and complement activity in the tick-bite area [47]. Subsequent experiments with *I. scapularis* engorged on *Borrelia burgdorferi*-infected mice demonstrated that Salp15 binds OspC, an outer-surface protein produced by *B. burgdorferi*, which facilitates transmission during tick feeding [38]. Immunization of mice with Salp15 resulted in 50% protection against *B. burgdorferi* infection, suggesting that the vaccination of hosts with the target molecules required for pathogen transmission may be used for disease prevention [48]. Recently, an mRNA vaccine, encoding for 19 salivary proteins from *I. scapularis* (including Salp15), was tested in guinea pigs, and a reduction in tick feeding and *B. burgdorferi* infection were confirmed [39].

Tick microbiome manipulation through host vaccination is a novel approach that has been recently investigated using an *I. ricinus*–mouse model [42]. Keystone bacteria family (Enterobacteriaceae) in the microbiome of *I. ricinus* were targeted through vaccination and the evaluation of nymph-feeding performance on vaccinated mice. High mortality during nymph feeding suggested that anti-tick microbiota vaccines may be used to evaluate the function of a specific taxon in tick microbiome, thus facilitating the identification of new targets for tick interventions and blocking the transmission of tick-borne pathogens [43].

In conclusion, since the publication of Allen and Humphreys in 1979 [1], anti-tick vaccine research has greatly advanced. Two commercial vaccines against the cattle tick *R. microplus* have been independently produced, and many new molecular targets from different tick species have been identified. Although anti-tick vaccine research has expanded to different tick species, *R. microplus* remains the most studied, as effective anti-tick vaccines against this tick would provide the most significant contribution to a reduction in the chemical products used to treat cattle raised in tick-endemic regions. Vaccines for the reduction in tick populations and blocking the transmission of pathogens causing human diseases are also needed. Developing anti-tick vaccines is challenging, but considering the experience with Bm86 vaccines, the availability of genomics and transcriptomics resources, and the newly reported tools for manipulation of the tick microbiome, effective anti-tick vaccines for targeting both ticks and tick-vectored pathogens are expected.

## Data Availability

Not applicable.
